# Human cytomegalovirus infection inhibits CXCL12- mediated migration and invasion of human extravillous cytotrophoblasts

**DOI:** 10.1186/1743-422X-9-255

**Published:** 2012-11-01

**Authors:** Jessica A Warner, Kevin J Zwezdaryk, Bonita Day, Deborah E Sullivan, Gabriella Pridjian, Cindy A Morris

**Affiliations:** 1 Department of Microbiology and Immunology, Tulane University School of Medicine, New Orleans, USA; 2 Physician-Scientist Program, Tulane University School of Medicine, New Orleans, USA; 3 Department of Obstetrics and Gynecology, Tulane University School of Medicine, 1430 Tulane Avenue, New Orleans, LA, USA

**Keywords:** Human cytomegalovirus, Placentation, Extravillous cytotrophoblasts, Chemokines, CXCL12, CXCR4 and CXCR7, Invasion

## Abstract

**Background:**

During the first trimester of pregnancy, a series of tightly regulated interactions govern the formation of a highly invasive population of fetal-derived extravillous cytotrophoblasts (EVT). Successful pregnancy is dependent on efficient invasion of the uterine wall and maternal spiral arteries by EVT. Dysregulated trophoblast invasion is associated with intrauterine growth restriction, birth defects, spontaneous abortion and preeclampsia. A number of soluble growth factors, cytokines, and chemokines modulate this process, fine-tuning the temporal and spatial aspects of cytotrophoblast invasion. In particular, the CXCL12/CXCR4 axis has been shown to specifically modulate cytotrophoblast differentiation, invasion, and survival throughout early pregnancy. Infection with human cytomegalovirus (HCMV) has been associated with impaired differentiation of cytotrophoblasts down the invasive pathway, specifically dysregulating the response to mitogens including epidermal growth factor (EGF) and hepatocyte growth factor (HGF). In this study, the effect of HCMV infection on the CXCL12-mediated migration and invasion of the EVT cell line SGHPL-4 was investigated.

**Results:**

Infection with HCMV significantly decreased secretion of CXCL12 by SGHPL-4 cells, and induced a striking perinuclear accumulation of the chemokine. HCMV infection significantly increased mRNA and total cell surface expression of the two known receptors for CXCL12: CXCR4 and CXCR7. Functionally, HCMV-infected SGHPL-4 cells were unable to migrate or invade in response to a gradient of soluble CXCL12 in transwell assays.

**Conclusions:**

Collectively, these studies demonstrate that HCMV impairs EVT migration and invasion induced by CXCL12. As HCMV has the ability to inhibit EVT migration and invasion through dysregulation of other relevant signaling pathways, it is likely that the virus affects multiple signaling pathways to impair placentation and contribute to some of the placental defects seen in HCMV-positive pregnancies.

## Background

Primary infection with human cytomegalovirus (HCMV) during the first trimester of pregnancy is associated with poor pregnancy outcomes, including intrauterine growth restriction (IUGR), birth defects, preeclampsia, and spontaneous abortion [1-3]. Specifically, HCMV has been shown to productively infect placental cytotrophoblasts. These cells undergo differentiation through a series of tightly regulated processes involving soluble growth factors, hormones and chemokines during the first trimester, resulting in an invasive subpopulation of cells termed extravillous cytotrophoblasts (EVT). EVT invade the uterine wall and remodel the spiral arteries within the endometrium and myometrium, producing a hybrid fetal-maternal vasculature necessary for the maintenance of pregnancy [4-7]. Inadequate trophoblast invasion impairs blood, oxygen and nutrient flow to the developing fetus, and can lead to shallow placentation and poor pregnancy outcomes for both mother and fetus [8-10]. Productive HCMV infection of cytotrophoblasts induces downregulation of cell surface adhesion molecules including VE-cadherin and α1β1 integrin, as well as the non-classical MHC molecule HLA-G, which may alter the immune response to infected cells [11,12]. Additionally, HCMV infection inhibits proliferation, matrix metalloproteinase (MMP) production, and invasion of extracellular matrix by the human first trimester-derived EVT cell line SGHPL-4, indicating that the virus blocks differentiation down an invasive pathway in EVT [13]. The molecular mechanism(s) by which HCMV inhibits EVT migration and invasion is not known.

The chemokine CXCL12, through interaction with the CXCR4 receptor, modulates differentiation and migration of a number of cell types, such as those in the central nervous system and in solid organ neoplasms including kidney, breast and ovarian cancers [14-19]. This chemokine also plays an integral role in EVT differentiation and invasion during early stages of placentation. CXCL12 induces proliferation and invasion in both cytotrophoblast cell lines and primary placental tissues [20-22]. Via interaction with CXCR4, CXCL12 mediates cytotrophoblast proliferation, MMP production, and invasion through basement membranes via activation of the mitogen-activated protein kinase (MAPK) pathway [20,21,23]. Recently, CXCL12 has been shown to bind to and activate a second receptor, CXCR7. Although G-protein coupled receptor activity has not been demonstrated for CXCR7, it appears to modulate the cellular response to CXCL12 via heterodimerization with CXCR4 as well as via induction of ligand sequestration during migration [24-28]. CXCR7, like CXCR4, is expressed in placental tissue; however, a specific role for this receptor in trophoblast function and placental development has not yet been defined [29,30]. Recent studies suggest that during the first trimester of pregnancy, cytotrophoblast-derived CXCL12 regulates the extent of EVT invasion by inducing expression of CD82 by decidual stromal cells, thereby preventing excessive invasion of the placenta into the uterine wall [31].

The aim of this study was to determine the effect of HCMV infection on cellular distribution and expression of CXCL12 and its receptors, CXCR4 and CXCR7, and on EVT migration and invasion in response to CXCL12.

## Results

### HCMV infection induces sequestration of endogenous CXCL12 in the extravillous cytotrophoblast cell line, SGHPL-4

**Figure 1:**
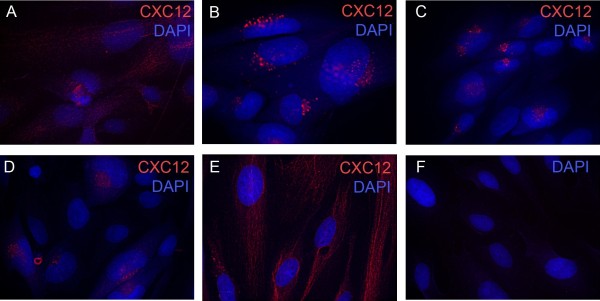
**SGHPL-4 cells express CXCL12 and HCMV infection induces perinuclear sequestration of CXCL12 in SGHPL-4 cells.** SGHPL-4 cells demonstrated positive staining for CXCL12 and mock-infected cells displayed diffuse staining for CXCL12 (**A**). SGHPL-4 cells were seeded on chamber slides and serum starved for 24 hours. They were infected with either laboratory (Towne and Toledo) or clinical (TRpM1a) isolates of HCMV for 24 hours, and then fixed and stained for CXCL12. In contrast, Towne (**B**), Toledo (**C**), and TRpMIA (**D**) were able to induce perinuclear sequestration of CXCL12. UV-inactivated Towne-GFP (**E**) was unable to induce chemokine sequestration. No significant background staining was seen in cells stained with secondary antibody and DAPI alone (**F**).

Immunofluorescence staining was performed to compare the cellular distribution of CXCL12 in mock- versus HCMV-infected cells. Serum-starved SGHPL-4 cells were either mock-infected or infected with HCMV isolated laboratory strains Towne (high passage) or Toledo (low passage), and the clinical strain TRpM1a, for 24 hours, at which point they were fixed and stained for CXCL12. As shown in Figure 1, infection with all three viral strains induced a stark alteration in the distribution of CXCL12. Mock-infected cells (Figure 1A) displayed diffuse staining for CXCL12. In contrast, in HCMV-infected cells (Figure 1B-D), CXCL12 was tightly clustered in a perinuclear staining pattern.

To assess whether viral gene expression is required for sequestration of CXCL12, SGHPL-4 cells were also infected with UV-inactivated Towne-GFP for 24 hours, at which point they were fixed and stained as above. No GFP expression was seen in cells infected with UV-inactivated HCMV, demonstrating a lack of viral gene expression (data not shown). UV-inactivated HCMV did not induce the perinuclear sequestration of CXCL12 (Figure 1E) seen after infection with wild-type virus. Staining with isotype-matched secondary antibodies alone served as a negative control (Figure 1F). These data indicate that expression of viral genes, rather than the activity of pre-packaged virion-associated proteins, is required for the altered distribution pattern of CXCL12 seen post-infection.

### Secretion of CXCL12 is decreased in HCMV-infected EVT

**Figure 2:**
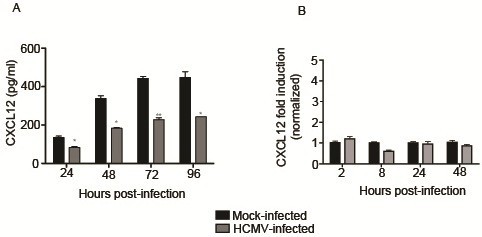
**HCMV infection decreases secretion of CXCL12 by SGHPL-4 cells but mRNA expression is unaffected.** Supernatants were collected from mock-infected or HCMV-infected cells and CXCL12 secretion was analyzed by ELISA (**A**). At all timepoints studied, HCMV-infected cells secreted significantly less CXCL12 into their culture medium as compared to mock-infected controls. RNA was isolated from mock-infected and HCMV-infected cells and quantitative real-time PCR was performed using primers specific for CXCL12 (**B**). There was no significant difference in mRNA expression between mock-infected and HCMV-infected cells at any timepoint analyzed. (*, p<0.05; **, p<0.01; ***, p<0.001).

To assess cellular secretion of CXCL12 following HCMV infection, cell culture supernatants were analyzed by ELISA (Figure 2A). Baseline secretion of CXCL12 in mock-infected cells increased between 24 and 72 hours in culture, at which point it declined. HCMV infection significantly decreased the concentration of CXCL12 in the supernatants at 24, 48, 72, and 96 hours post-infection (p<0.05).

### Expression of CXCL12 at the mRNA level is unchanged by HCMV infection

Serum-starved SGHPL-4 cells were infected with HCMV or UV-inactivated virus and RNA lysates were harvested at 2, 8, 24, or 48 hours post-infection with HCMV. Quantitative real-time PCR was performed using specific primers to CXCL12 (Table 1). All threshold values were normalized to the 36B4 ribosomal housekeeping gene. Infection with HCMV did not significantly alter mRNA expression of CXCL12 at any timepoint analyzed (Figure 2B). UV-inactivated HCMV also did not affect mRNA expression of CXCL12 (data not shown).

**Table 1:** Real-time PCR primer sequences

**Gene**	**Sequence**	**Accession number**
CXCL12	Sense: 5^′^-ACT-GGG-TTT-GTG-ATT-GCC-TCT-GAA-3^′^	NM00117813
	Antisense: 5^′^-GGA-ACC-TGA-ACC-CCT-GCT-GTG-3^′^	
CXCR4	Sense: 5^′^-TTG-TGG-GTG-GTT-TGT-GTT-CCA-3^′^	NM003467
	Antisense: 5^′^-CTG-TGG-TCT-TGA-GGG-CCT-TG-3^′^	
CXCR7	Sense: 5^′^-GGC-TAT-GAC-ACG-CAC-TGC-TAC-A-3^′^	NM020311
	Antisense: 5^′^-TGG-TTG-TGC-TGC-ACG-AGA-CT-3^′^	
36B4	Sense: 5^′^-TGG-AGA-CGG-ATT-ACA-CCT-TC-3^′^	NM001002
	Antisense: 5^′^-CTT-CCT-TGG-CTT-CAA-CTT-TAG-3^′^	

### HCMV infection alters expression of CXCR4 and CXCR7 at the cell surface

**Figure 3:**
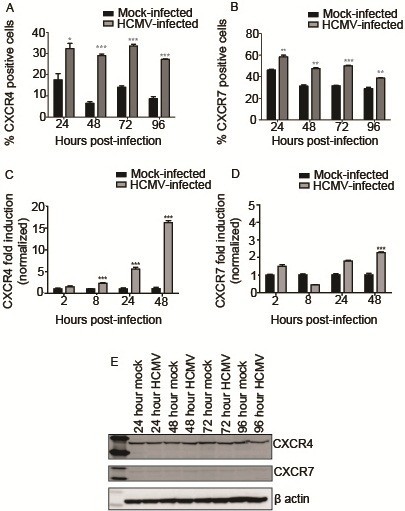
**HCMV infection alters expression of CXCR4 and CXCR7.** SGHPL-4 cells were mock-infected or infected with HCMV, and then CXCR4 and CXCR7 receptor expression was determined. HCMV infection induced upregulation of cell surface staining for CXCR4 (**A**) and CXCR7 (**B**) at all time points studied. (*, p<0.05; **, p<0.01; ***, p<0.001). Total RNA was harvested from mock-infected or HCMV-infected SGHPL-4 cells, and analyzed by real-time PCR using primers specific for CXCR4 and CXCR7. HCMV infection induced a significant upregulation of CXCR4 mRNA expression (**C**) at 8, 24, and 48 hours post-infection. A modest but significant upregulation of CXCR7 mRNA expression was seen at 48 hours post-infection (**D**). Protein lysates from mock-infected and HCMV-infected cells were analyzed by Western blot for CXCR4 and CXCR7 expression. No differences in absolute protein levels were seen at any time point analyzed (**E**).

Given perinuclear sequestration and decreased secretion of CXCL12 following HCMV infection of SGHPL-4 cells, the abundance and distribution of the CXCL12 receptors, CXCR4 and CXCR7, were also analyzed. Surface expression of CXCR4 and CXCR7 was assessed by flow cytometry. Mock-infected or HCMV-infected SGHPL-4 cells were harvested and surface-stained for these chemokine receptors using antibodies directly conjugated to phycoerythritin (PE). As shown in Figure 3A-B, infection with HCMV significantly increased the percentage of cells expressing CXCR4 and CXCR7 on the cell surface at all timepoints analyzed. The mean fluorescence intensity (MFI) for CXCR4, corresponding to the number of molecules of each receptor on the cell surface, was increased at all timepoints analyzed, whereas the MFI for CXCR7 was increased only at 72 and 96 hours post-infection (data not shown).

### CXCR4 and CXCR7 mRNA expression is increased by HCMV infection

Infection with HCMV induced upregulation of CXCR4 mRNA expression (p<0.001) at 8, 24, 48, and 96 hours post-infection (Figure 3C). Peak upregulation of CXCR4 mRNA expression was seen at 96 hours, with a 16-fold increase in expression between HCMV-infected and mock-infected samples. Although a statistically significant (p<0.001) upregulation of CXCR7 mRNA expression was seen at 48 hours post-infection, barely a two-fold change was detected, (Figure 3D). Infection with UV-inactivated HCMV did not alter the mRNA expression of CXCR4 or CXCR7 at any timepoint analyzed, indicating that viral gene expression is required for these findings (data not shown).

### Absolute protein levels of CXCR4 and CXCR7 are unchanged by HCMV infection

Given the upregulation of surface and mRNA expression of CXCR4 and CXCR7 following HCMV infection, it seemed likely that total protein levels may likewise be increased. Serum-starved SGHPL-4 cells were mock-infected or infected with wild-type HCMV for 24, 48, 72, or 96 hours. Protein lysates were harvested at appropriate time points post-infection and equal amounts of total protein were subjected to Western blotting for CXCR4 and CXCR7. A single band corresponding to CXCR4 was detected in all samples. Surprisingly, the band intensity for both receptors was not altered at any time point post-infection (Figure 3E).

### HCMV infection blocks CXCL12-induced migration and invasion

To determine whether HCMV infection affects CXCL12-induced EVT migration and invasion, the functional response of HCMV-infected cells to exogenous CXCL12 was assessed using a quantitative FluoroBlok assay that has been used widely to characterize cytotrophoblast migration and invasion to chemotactic stimuli [13,32,33]. Recombinant human epidermal growth factor (EGF) (10 ng/ml) was used routinely as a positive control for each assay, as SGHPL-4 cells significantly migrate and invade in response to EGF, while infection with HCMV abrogates this response (data not shown) [13].

**Figure 4:**
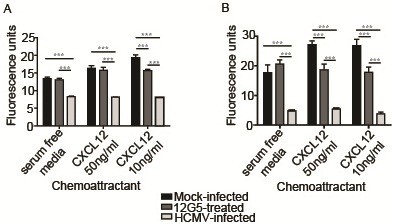
**HCMV infection blocks the ability of SGHPL-4 cells to migrate or invade toward CXCL12.** Serum-starved SGHPL-4 cells were mock-infected or infected with Towne HCMV (MOI of 1) for 24 hours. Mock-infected cells were also pretreated with a neutralizing antibody against CXCR4 (12G5) prior to the assay. The cells were loaded into Fluoroblok inserts with 8 μm pores and chemoattractants (CXCL12 or EGF) were loaded into the lower chamber. Migration was allowed to proceed for 6 hours, whereas invasion assays were incubated for 24 hours, at which point they were labeled with Calcein AM and 10X images were acquired. HCMV infection significantly impaired migration (**A**) and invasion (**B**) toward CXCL12 at both 50 ng/ml and 10 ng/ml (p<0.0001). Although antibody-mediated (12G5) neutralization of CXCR4 significantly decreased migration (p<0.0001) and invasion (p<0.0001) toward CXCL12, its effect was not as pronounced as that seen following infection with HCMV. (***, p<0.001).

CXCL12 induced migration of SGHPL-4 cells to a greater extent than that observed using serum-free culture medium alone (negative control) (Figure 4A). HCMV infection blocked migration toward recombinant human CXCL12 at both 50 ng/ml and 10 ng/ml (p<0.001) to the level seen in negative control wells containing serum-free culture medium alone (Figure 4A). The specific role of CXCR4 in this result was investigated using a neutralizing antibody directed against the receptor (clone 12G5) or an isotype-matched mouse anti-human IgG control. Data not shown) interestingly, the impaired migration seen following HCMV infection was more dramatic than that seen following neutralization of CXCR4. In fact, 12G5 was unable to significantly inhibit migration toward CXCL12 at a concentration of 50 ng/ml, although it was able to inhibit migration toward 10 ng/ml of the recombinant chemokine (p<0.001). Control IgG did not have a statistically significant impact on migration (data not shown).

CXCL12 also induced invasion of SGHPL-4 cells versus cells incubated with serum-free medium alone (Figure 4B). SGHPL-4 cell invasion through Matrigel toward CXCL12 at both 50 ng/ml and 10 ng/ml was also significantly impaired following HCMV infection (p<0.001), again recapitulating the weak background migration toward serum-free culture medium. In contrast to the migration assays, pre-treatment with 12G5 was able to significantly inhibit invasion toward both 50 ng/ml and 10 ng/ml of CXCL12 (p<0.001), however, not to the level seen in HCMV-infected cells. Once again, control IgG did not have a significant effect on invasion (data not shown).

## Discussion

The early events in placentation, including proliferation of villous cytotrophoblasts, as well as their adoption of an invasive phenotype, are inhibited by infection with HCMV [13,34-38]. This ubiquitous human pathogen, which generally causes subclinical disease in immunocompetent patients, can have devastating consequences for both the mother and developing fetus when primary infection occurs in the first trimester of pregnancy [1-3]. Previous studies have shown that HCMV impairs the response of EVT to soluble EGF in terms of their ability to proliferate, migrate and invade [13]. However, the effect of HCMV on the response of EVT to other soluble mediators has largely been unexplored. The aim of this study was to specifically address the effect of HCMV infection on the cellular localization and expression of the CXCL12 chemokine and its receptors, CXCR4 and CXCR7, in the extravillous cytotrophoblast cell line SGHPL-4 and to determine whether HCMV affects EVT migration and invasion.

Immunofluorescence staining for CXCL12 in HCMV-infected SGHPL-4 cells demonstrated clear and distinct perinuclear accumulation of the chemokine. CXCL12 sequestration was dependent on viral gene expression, as perinuclear accumulation of the chemokine was not seen after infection with UV-inactivated virus. Additionally, secretion of CXCL12 into culture medium was inhibited by infection with HCMV, whereas mRNA expression of CXCL12 was unaffected by viral infection in SGHPL-4 cells. This suggests that the decrease in CXCL12 secretion seen following HCMV infection is a function of decreased release or degradation of preformed chemokine, rather than altered generation of new transcripts or protein. Colocalization studies are needed to determine the precise subcellular localization of CXCL12 in HCMV-infected cells to more clearly delineate whether there is an inhibition of secretion or an increase in degradation of the chemokine.

The effect of HCMV infection on expression of the CXCL12 receptors, CXCR4 and CXCR7, was also investigated. Infection of SGHPL-4 cells with HCMV induced upregulation of both receptors to the cell membrane. HCMV infection also upregulated mRNA expression of both CXCR4 and CXCR7; however, protein expression levels of both chemokine receptors were unaffected by HCMV infection. This finding may be due to increased protein degradation through proteasome and/or autophagosome activation, viral hijacking of the cellular translational machinery, stability or rapid turnover of mRNA, CXCL12 endocytic trafficking to lysosomes for degradation and/or other possible posttranscriptional regulatory mechanisms. Upregulation of cell surface CXCR7 expression in parallel to that seen for CXCR4 was not surprising, since these receptors were recently shown to heterodimerize [28]. However, the precise function of CXCR7 in SGHPL-4 cells (and in cytotrophoblasts in general) remains to be elucidated. If CXCR7 in fact acts as a chemokine sink, upregulation of this receptor to the cell surface may mediate sequestration of CXCL12 following HCMV infection [26,27,29,39].

Despite recruitment of CXCR4 and CXCR7 to the cell membrane, both migration and invasion toward CXCL12 were almost completely inhibited following HCMV infection. These results indicated that despite increased cell surface receptor expression, HCMV-infected cells were unable to respond to a chemotactic gradient of CXCL12. Blockade of CXCR4 function with a neutralizing antibody (12G5) partially inhibited migration and invasion toward CXCL12, but to a lesser extent than the inhibition seen following HCMV infection, suggesting that both CXCR4 and CXCR7 may mediate migration and invasion in response to CXCL12. In general, upregulation of cell surface expression of CXCR4 is associated with a pro-migratory phenotype. Treatment of adult neuronal stem cells or human mesenchymal stem cells with cytokines including IL-3, IL-6, and IL-10 induces cell surface expression of CXCR4, which correlates with increased cellular migration [40,41]. Additionally, the macrolide antibiotic erythromycin, which demonstrates potent anti-inflammatory effects, has been shown to upregulate cell surface expression of CXCR4 on microvascular endothelial cells, promoting their migration into sites of tissue injury [42]. Therefore, the finding that CXCL12-induced migration and invasion were decreased in spite of upregulated cell surface expression of CXCR4 and CXCR7 in HCMV-infected SGHPL-4 cells is somewhat surprising. Given the decreased secretion of CXCL12 following HCMV infection of SGHPL-4 cells, it is possible that HCMV-infected cells upregulate cell surface and mRNA expression of CXCR4 and CXCR7 as an attempt to compensate for lack of or dysregulated autocrine signaling.

Ultimately, these studies demonstrate that HCMV inhibits CXCL12-induced EVT migration and invasion that correlates with sequestration and reduced secretion of the chemokine CXCL12. The molecular mechanism(s) by which HCMV inhibits cytotrophoblast migration and invasion during placentation is not known but likely involves, in general, the virus hijacking the cellular machinery and thus affecting cellular morphology, cytoskeletal arrangement, endosomal trafficking and invadosome formation.

## Conclusion

Collectively, these studies demonstrate that CXCL12 is sequestered during HCMV infection in placental cells and that HCMV inhibits EVT migration and invasion. Whether the ability of HCMV to inhibit EVT invasive properties is due to dysregulation of the CXCL12/CXCR4 signaling axis remains undetermined. HCMV dysregulation of placental function associated with pregnancies complicated by primary HCMV infection is mediated in part through a mechanism that involves inhibition of EVT migration and invasion induced by CXCL12. Since HCMV similarly inhibits EVT migration and invasion induced by other factors present at the fetal-maternal interface, HCMV may likely be affecting cellular function to abrogate EVT invasiveness. Future studies may elucidate convergent pathways that regulate cytotrophoblast differentiation into an invasive phenotype.

## Materials and methods

### Cells

The human extravillous trophoblast cell line SGHPL-4 (kindly provided by Drs. Guy Whitley and Judith Cartwright, St. George’s University, London, England) was used for these studies. This cell line was derived from first-trimester chorionic villous tissue immortalized with SV40 large T antigen. These cells have been shown to share many characteristics with first-trimester primary EVT, including expression of cytokeratin-7, HLA-G, hCG, and hPL when cultured on an artificial extracellular matrix (Matrigel) [43]. These cells are permissive for the full replication cycle of human cytomegalovirus [13,44]. SGHPL-4 cells were cultured in Ham’s F10 medium, supplemented with 10% fetal bovine serum, 2 mM L-glutamine, 100 U/ml penicillin G, and 100 mg/ml streptomycin (all from Invitrogen, Carlsbad, CA) at 37°C in 5% CO_2_. For serum starvation, the media was replaced with Ham’s F10 prepared as above but with reduced FBS (0.5%).

HCMV was propagated in human foreskin fibroblast cells (HFF; ATCC, Manassas, VA). These cells were maintained in DMEM supplemented with 10% FBS, 2 mM L-glutamine, 100 U/ml penicillin G, and 100 mg/ml streptomycin (all from Invitrogen) at 37°C in 5% CO_2_.

### Viral strains and propagation

Several viral strains were used for these studies. Towne-GFP (an attenuated but well-characterized laboratory isolate) and TRpM1a (a clinical isolate) were kindly provided by Dr. Dan Streblow (Oregon Health Sciences University, Beaverton, OR). Other viral strains (Toledo and Towne) were purchased from ATCC. Total virus was prepared from cell culture supernatants of infected HFFs as previously described [13]. Briefly, infected HFFs were incubated at 37°C in 5% CO_2_ in DMEM containing 10% FBS until 100% of the cells demonstrated cytopathic effect (CPE). Regular feeding of the cells was then ceased and the cells were incubated for an additional 4–5 days, at which point the cells were easily detached from the plastic. Supernatants were collected and clarified by centrifugation for 10 minutes at 2500 rpm at 4°C. The virus was aliquoted and frozen at −80°C and then stored in liquid nitrogen. Viral titers were calculated by staining for the immediate early antigens of HCMV 24 hours post-infection.

### HCMV infections

All HCMV infections were carried out as follows. Briefly, SGHPL-4 cells were seeded on tissue culture plates and allowed to adhere for at least 24 hours at 37°C. The cells were then washed with 1X Dulbecco’s phosphate buffered saline (DPBS, Invitrogen) and serum-starved in Ham’s F10 medium containing 0.5% FBS for an additional 24 hours. HCMV was diluted in serum-free Ham’s F10 in a minimal volume for the surface area to be infected. The cells were incubated with virus at 37°C for 90 minutes with occasional rocking. Unbound virus was removed by aspiration and fresh Ham’s F10 containing 0.5% FBS was added to the infected cells. For each experiment, control cells were mock-infected using equivalent volumes of media.

HCMV was UV-inactivated as previously described [13]. Briefly, virus thawed on ice was spread onto a 10-cm plate and cross-linked four times at 426 mJ, with gentle mixing after each cycle. The virus was then diluted and used for infections as described above.

### Antibodies and reagents

For immunofluorescence, the following primary antibodies were used: mouse anti-human CXCL12 (R&D Systems, Minneapolis, MN, MAB350). Detection of HCMV immediate early (IE) gene expression was performed using mouse anti-human cytomegalovirus IE 1/2 (Millipore, Billerica, MA, MAB810). Isotype-matched secondary antibodies conjugated to either Alexa-488, Alexa-555, or Alexa-594 (Invitrogen) were used as indicated. For flow cytometric analysis of chemokine receptor expression, mouse anti-human CXCR4 (eBioscience, San Diego, CA, 12–9999) and mouse anti-human CXCR7 (Biolegend, San Diego, CA, 331104) antibodies directly conjugated to phycoerythritin (PE) were used.

For Western blotting, the following antibodies were used: rabbit anti-human CXCR4 (Abcam, ab2074), rabbit anti-human CXCR7 (Abcam, ab72100), and rabbit anti-human beta actin (Abcam, ab8227). Appropriate secondary antibodies conjugated to horseradish peroxidase (HRP) were used for detection (1:5000, KPL, Gaithersburg, MD).

Recombinant CXCL12 was purchased from R&D Systems and reconstituted in DPBS supplemented with 0.1% bovine serum albumin. Neutralization of CXCR4 activity was obtained with mouse anti-human CXCR4 antibody (clone 12G5, Abcam ab21555) diluted to a final concentration of 10 μg/ml in serum-free Ham’s F10 medium.

### ELISA analysis

SGHPL-4 cells were infected with HCMV at an MOI of 1 and supernatants were harvested at appropriate timepoints post-infection. The supernatants were spun at 2500 rpm to pellet any cellular debris, aliquoted, and frozen at −80°C prior to analysis.

Secretion of CXCL12 into cell culture supernatants was analyzed by enzyme-linked immunosorbent assay (ELISA, R&D Systems, SDF-1α Quantikine Immunoassay kit) per the manufacturer’s instructions. ELISA plate absorbance was read at 450 nm with correction at 540 nm using a μQuant Universal Microplate Spectrophotometer (BioTek Instruments; Winooski, VT). The data were normalized to a standard curve prepared with serial dilutions of recombinant CXCL12.

### Immunofluorescence

SGHPL-4 cells were plated on 4-well chamber slides coated with collagen type IV (BD Biosciences), and then mock-infected or infected with HCMV as outlined above. At appropriate timepoints, the cells were fixed with 2% methanol free paraformaldehyde (Ted Pella, Inc. Redding, CA) for 20 minutes at room temperature. Free aldehydes were quenched by incubation with 50mM NH_4_Cl for 10 minutes, and the cells were permeabilized with 0.1% Triton-X 100 in DPBS for 8 minutes. The slides were blocked with DPBS supplemented with 5% BSA and 0.05% Triton-X 100 for 1 hour at room temperature. Primary antibodies were diluted in DPBS supplemented with 1% BSA and 0.05% Triton-X 100 for 1 hour. After extensive washing, secondary antibodies conjugated to Alexa-488, Alexa-555, or Alexa-594 (Invitrogen) were added and nuclei were counterstained with 4^′^,6-diamidino-2-phenylindole,dihydrocholoride (DAPI, Invitrogen). The slides were then washed with DPBS and mounted with Prolong Antifade mounting medium (Invitrogen) and covered with glass coverslips. For each experiment, wells stained with secondary antibodies alone were included as controls for background fluorescence. Slides were imaged using a Zeiss Axio Plan II microscope (Thornwood, NY) and images were deconvolved using SlideBook 5.0 Intelligent Imaging Software (Intelligent Imaging Innovations; Denver, CO).

### Flow cytometry

Cell surface expression of CXCR4 and CXCR7 was analyzed by flow cytometry. SGHPL-4 cells grown in 6-well plates were serum-starved for 24 hours at 37°C. The cells were then infected with Towne HCMV at an MOI of 1 and incubated for an additional 24, 48, 72, or 96 hours. The samples were blocked in DPBS containing 2% FBS for 20 minutes on ice prior to incubation in blocking solution containing antibodies to CXCR4 and CXCR7 directly conjugated to PE for 30 minutes on ice in the dark. After washing, the samples were fixed in 4% methanol-free formaldehyde (Ted Pella, Inc.) for 20 minutes in the dark, and then washed with DPBS. The samples were stored in DPBS containing 0.1% BSA and 0.1% NaN_3_ at 4°C until they were run on the flow cytometer. The samples were analyzed using a BD FACSCalibur on CellQuest Pro software (BD Biosciences, San Jose, CA). Unstained control cells and cells stained with PE-conjugated isotype controls were used to set the acquisition controls and voltages. The data were analyzed using CellQuest Pro software (BD Biosciences).

### Western blotting

SGHPL-4 cells were plated on 10-cm plates and grown to near confluency, then serum-starved in Ham’s F10 containing 0.5% FBS for 24 hours. For long-term HCMV infection studies, the cells were infected with HCMV at MOI of 1 for 90 minutes at 37°C, at which point unbound virus was removed and the cells were incubated in Ham’s F10 containing 0.5% FBS for 24–96 hours. Mock-infected controls were included for each time point.

Protein lysates were harvested as previously described [13,45]. The plates were washed in cold DPBS and the cells were lysed with RIPA buffer containing a proteinase inhibitor cocktail (PIC), two phosphatase inhibitors (PhIC I and II) and 1mM phenylmethanesulfonylfluoride (PMSF; all from Sigma-Aldrich; St. Louis, MI). The lysates were sheared and. cellular debris was removed. Total protein concentrations were estimated with the Micro BCA protein assay kit (Thermo Fisher; Rockford, IL).

Total protein (25 μg per sample) were separated on 4-12% NuPage SDS-polyacrylamide gels (Invitrogen). Proteins were transferred by blotting to PVDF membranes (Invitrogen) and were blocked in 5% non-fat dry milk (NFDM) in Tris-buffered saline containing 0.1% Tween-20 (TBST) prior to incubation with primary antibody overnight at 4°C. After extensive washing, the blots were incubated with secondary antibodies (goat anti-mouse IgG, rabbit anti-goat IgG or goat anti-rabbit IgG, 1:5000, KPL Protein Research Products) for 1 hour at room temperature. Antigen-antibody complexes were detected using an enhanced chemiluminescence system (Thermo Fisher) and digital images of blots were acquired using an LAS4000 imager (Kodak, Rochester, NY). Blots were subsequently stripped in Restore Plus Western Blot Stripping Buffer (Thermo Fisher) and reprobed using a rabbit polyclonal antibody directed against human β-actin (Abcam) as a loading control.

### Quantitative real-time PCR

Total RNA was isolated from serum-starved SGHPL-4 cells that had been mock-infected, infected with UV-inactivated HCMV, or infected with the Towne HCMV strain. RNA was isolated using the Qiagen RNeasy Mini kit according to the manufacturer’s instructions (Qiagen, Valencia, CA). Isolated RNA was treated with DNase I (Ambion, Inc.; Austin, TX) and concentration was measured using a NanoDrop Spectrophotometer (Thermo Scientific). Total RNA (1 μg) was reverse transcribed with iScript cDNA Synthesis Kit (Biorad; Hercules, CA) as per the manufacturer’s instructions.

Quantitative real-time PCR was performed using the iCycler Real-Time PCR Detection System (BioRad). Specific primer pairs used in these aims are listed in Table 1. PCR cycling conditions were as follows: 1 denaturing step at 95°C for 3 minutes, 40 cycles of 95°C for 15 seconds followed by 60°C for 30 seconds, 1 cycle at 95°C for 1 minute, and 1 cycle of 60°C for 1 minute. Following the PCR reaction, melt curve reactions were performed to confirm the specificity of each primer pair and exclude the presence of primer dimers from the reaction. The melt curve was performed via 80 cycles beginning at 55°C and incrementally increasing by 0.5°C every 30 seconds. Relative quantitation was determined using the comparative C_T_ method with data normalized to 36B4 and calibrated to the average C_T_ of mock-infected control at the specified time point.

### Migration and invasion assays

Cellular migration and invasion were determined using the quantitative FluoroBlok assay as previously described [13]. Serum-starved SGHPL-4 cells were mock- infected or infected with Towne HCMV as described above. 24 hours post-infection, the cells were trypsinized and loaded into FluoroBlok inserts with 8 μm pores (BD Biosciences). To assess the role of CXCR4 on migration and invasion toward CXCL12, some samples were pre-treated with 12G5 (a neutralizing antibody against CXCR4) or a mouse IgG isotype control, both at 10 μg/ml, for 30 minutes prior to the beginning of the migration or invasion assay.

For invasion assays, the inserts were pre-coated with growth factor reduced Matrigel (BD Biosciences) diluted 1:10 in serum-free Ham’s F10 and the Matrigel was allowed to solidify for 1 hour at 37°C. For migration assays, the inserts were left uncoated. Each well was loaded with 2.5x10^5^ cells in a total volume of 200 μl of serum-free medium. The lower wells of the chamber were loaded with serum-free culture medium containing varied doses of recombinant CXCL12. Human EGF (10 ng/ml) was used as a positive control for all assays and serum-free medium alone was used as a negative control. Invasion assays were allowed to proceed for 24 hours whereas migration assays were incubated for 6 hours.

At the end of the experimental time period, the plates were removed from the incubator and any cells remaining on top of the insert were removed by aspiration. The cells on the lower surface of the inserts were then fluorescently labeled with Calcein AM (Invitrogen). The plates were then incubated at 37°C for an additional hour, which allowed for fluorescent labeling of the invasive cells on the lower surface of the membrane. Four 10X pictures of each well were taken using a Nikon TE300 inverted epifluorescent microscope (Olympus Optical Company, Lewisville, TX) and the mean fluorescence per image was determined using ImageJ software (NIH, Bethesda, MD).

### Statistical analysis

The data are presented as mean ± standard error of the mean [46]. Data were compared to negative control samples and each other by one-way analysis of variance (ANOVA) followed by Dunnett’s multiple comparisons post hoc test (GraphPad Prism, Version 5, La Jolla, CA). For experiments with only two groups, data were analyzed by one-tailed unpaired t test. Statistical significance is denoted on figures with asterisks (*; p<0.05, **; p<0.01, ***; p<0.001).

## Competing interests

The authors declare that they have no competing interests.

## Authors’ contributions

JW performed all assays, statistical analysis, and drafted the manuscript. DS and KZ assisted with study design and edited the manuscript. BD performed immunofluorescent imaging and assisted with figure design. GP assisted with study design and reviewed the manuscript. CM conceived the study and revised the manuscript. All authors read and approved the final manuscript prior to submission.
